# Willingness of patients with chronic disease in rural China to contract with family doctors: implication for targeting characteristics

**DOI:** 10.1186/s12875-021-01553-2

**Published:** 2021-10-14

**Authors:** Jie Li, Jie Li, Peipei Fu, Yan Chen, Xue Tang, Zhixian Li, Shijun Yang, Chen Yan, Wenjuan Li, Zhen Gui, Chengchao Zhou

**Affiliations:** 1 Centre for Health Management and Policy Research, School of Public Health, Cheeloo College of Medicine, Shandong University, Jinan, 250012 China; 2 NHC Key Lab of Health Economics and Policy Research, Shandong University, Jinan, 250012 China; 3 School of Public Health, Wannan Medical College, Wuhu, 241002 China; 4 Department of Health Economics, School of Public Health, Fudan University, Shanghai, 200032 China

**Keywords:** Contract willingness, Chronic conditions, Family doctor, Family doctor contract services, Andersen model

## Abstract

**Background:**

Rural residents with chronic conditions have a stronger need for health services, which should make using family doctor contract services a priority. This study aimed to evaluate the rate of willingness among rural residents with chronic conditions to contract with family doctors and examine its determinants.

**Methods:**

A cross-sectional study was conducted from May, 2018 to June, 2018 in Shandong Province in China. A total of 769 rural unsigned residents with chronic conditions were included in the analysis. Using the Andersen model as the theoretical framework, logistic regression models were chosen to analyse the factors associated with willingness to contract with family doctors.

**Results:**

This study found that the rate of willingness to contract with family doctors among chronic patients in rural Shandong was 46.7%. A higher willingness was observed in those living a further distance from the village clinic (more than 600 m: OR = 1.85, 95%CI =1.17–2.93), having received publicity for family doctor contract services (OR = 1.71, 95% CI = 1.06–2.76), reporting need for utilizing a chronic disease management program (OR = 3.36, 95% CI = 2.20–5.23), and reporting need for higher medical insurance reimbursement (OR = 1.91, 95% CI = 1.28–2.83).

**Conclusions:**

The prevalence of contract willingness was relatively low among unsigned rural residents with chronic conditions in rural Shandong, China. The need factors were powerful factors affecting their willingness to contract with family doctors. The government should therefore strengthen targeted publicity and education to rural residents with chronic conditions and provide targeted healthcare services, such as chronic disease management programs and medical services with higher reimbursement rates, to promote their willingness to contract with family doctors.

**Supplementary Information:**

The online version contains supplementary material available at 10.1186/s12875-021-01553-2.

## Introduction

The increasing morbidity of chronic noncommunicable diseases resulting from the rapid ageing population is a great challenge for the healthcare system. In China, the prevalence of hypertension and diabetes in adults is 27.5 and 11.9%, respectively, in 2020 [1]. Strengthening the primary health care (PHC) system is an important measure to address this challenge. The PHC system has contributed substantially to a reduction in the burden of diseases [2]. China has introduced several policies to build integrated delivery systems based on the PHC to prevent and manage chronic diseases [3]. Family doctor contract services (FDCS) were one of the policies, they were adopted as a fundamental healthcare policy in 2009 [4] and extended to a large scale in 2016.

FDCS was launched in Shandong Province in 2016 [5], targeting the entire population and focusing on priority populations, such as the elderly, pregnant women, and patients with hypertension, diabetes, and other chronic diseases. FDCS aims to provide residents with proactive, continuous, and comprehensive primary health care by establishing stable partnerships between community residents and family doctors [4]. In addition, residents who contract with family doctors can obtain more benefits, such as access to chronic disease management programs, the green channels in referrals to specialists, and higher medical insurance reimbursement rates by visiting family doctors. Residents can choose their family doctor voluntarily and free to sign contracts. The contracts generally last for 1 year, and the residents can change the contracted doctors after the contract period expires.

The positive effects of FDCS have been reported in previous studies [6, 7]. FDCS can improve people’s access to primary health care and improve the health of community residents [8]. It also helps residents with chronic diseases to improve their health-related awareness, self-management behaviours, and treatment compliance. One study from China indicated [9] that approximately 80.79% of contracted patients had implemented noncommunicable diseases self-management, while only 55.57% of noncontracted patients did so. Another study [10] found that the blood pressure and blood control rates for the contracted group were higher than those of their counterparts, reaching up to 85.6 and 72.7% respectively. Therefore, residents with chronic conditions should make using family doctor contract services a priority.

Improving patients’ willingness to contract with family doctors is significant for their health. However, previous study showed that distrust in the quality of primary care and misunderstandings about family doctors are key barriers to signing contracts [11]. In addition, some studies have indicated a lower willingness to contract with family doctors among the general population [12–14], and the reasons mainly include poor access to regular medical care and poor publicity of FDCS. Unfortunately, existing studies about residents’ willingness to contract with family doctors mainly focus on the general population, and little is known about this topic among patients with chronic diseases.

To remedy this gap, this study aimed to evaluate the rate of willingness to contract with family doctors among rural patients with chronic conditions, and explore the factors associated with their willingness, to provide essential information to promote FDCS among patients with chronic diseases in rural Shandong, China.

### Conceptual framework

The Anderson model [15, 16] is a patient-based behavioural model that is widely used in studies on health service utilization. It postulates that health care utilization is determined by predisposing factors (age, sex, race, social class), enabling factors (socioeconomic factors, insurance), and need factors (illness-related factors). Currently, it is also used in the study of older people’s intended use of services [17–22]. Based on a previous study [23], we used the Anderson model to guide our current study (Appendix Fig. 1).

A range of predictors that may explain residents’ willingness to contract with family doctors have been identified in previous studies, such as 1) predisposing characteristics, including age, gender, educational level, and employment status [24–26]; 2) enabling resources, including marital status, financial condition, health insurance, distance to the village clinic, awareness of FDCS, and publicity of FDCS [12, 24]; and 3) need factors, including self-rated health status and the number of chronic diseases [26, 27]. Although the Andersen model includes “need factors”, limited attention has been given to need factors [28]. A previous study indicated that residents contracted with their family doctors based mainly on their real health care needs [29, 30]. Therefore, it is necessary to consider more need factors. In our study, we add three need factors related to chronic patients’ willingness into the Andersen model: need for utilizing referrals to specialists, need for utilizing chronic disease management programs and need for higher medical insurance reimbursement rates.

## Methods

### Study populations and sampling

A cross-sectional survey was conducted in three cities in Shandong Province from May to June 2018. Located in the northeast, central, and west of Shandong Province, Binzhou, Zibo, and Liaocheng were included in this study, which represent the medium, high, and low levels of economic status according to the GDP per capita (2018), respectively. A multistage stratified cluster sampling method was used to select participants, and all sampling processes followed strict randomization principles. First, we selected two counties from each city. Then, within each county, five townships were selected and six villages in each township were chosen. Finally a total of 2979 rural residents were recruited for the survey. According to our research purposes, only participants who: 1) had chronic diseases, 2) contracted with family doctors, and 3) had questionnaires that were without logical errors, were included. A total of 769 eligible questionnaires were included in the final analysis (Appendix Fig. 2).

All the participants were interviewed face-to-face using a structured questionnaire by trained interviewers. To obtain complete and accurate data, we excluded participants who could not answer independently and with hospital-diagnosed psychiatric disorders. Only one member of a family (generally the main income earner or the elderly) was recruited in this study.

### Measures

Contract willingness was measured by a question for unsigned rural residents with chronic diseases: “Family doctor contract services is a program that can provide people with primary and continuing health care services after contracting with family doctors. It is voluntary. Would you contract with a family doctor?” (yes, no).

Predisposing characteristics were age (64 or below, 65 or above), gender (female, male), educational level (illiteracy; primary school; junior middle school; senior high school or above), and employment status (unemployed, employed). As people with chronic diseases are mostly middle-aged and elderly people, age was divided into two groups by 65 years old.

Enabling resources were marital status (married, unmarried), health insurance (none, have), household annual income, distance to the village clinic, awareness of FDCS (never heard about it, know little about it, know much about it), and publicity of FDCS (yes, no). Awareness of FDCS was measured with the question “Do you know about the family doctor contract services?”. The publicity of FDCS was measured with the question “As far as you know, have you ever received publicity about family doctor services?”

Need factors included self-reported health status (good, general, bad), the number of chronic diseases (1, ≥ 2), need for utilizing referrals to specialists (yes, no), need for utilizing chronic disease management program (yes, no), and need for higher medical insurance (MI) reimbursement rates (yes, no). For the number of chronic diseases, using a yes-or-no format, participants were asked to report nine chronic diseases (e.g, hypertension, diabetes mellitus, cardiovascular and cerebrovascular diseases, respiratory disease). A summated score was used for the analysis, with a high score indicating more chronic conditions. The need for utilizing referrals to specialists was measured with the question “If a family doctor contract service can help your referrals to specialists, would you like to use it?”. Need for utilizing chronic disease management program was measured with the question “If family doctor contract service can help your chronic disease management, would you like to use it?”. The need for higher medical insurance (MI) reimbursement rates were measured with the question “If a family doctor contract service can increase the proportion of medical insurance reimbursement for medical services, would you like to use it?”

### Ethical considerations

This study was approved by the Ethical Committee of Shandong University School of Public Health. Our survey was voluntary, and residents could refuse to participate. All participants (the guardian of illiterate participants) provided informed consent before the face-to-face interview. The data used in this study were anonymized before use. All procedures performed in this study involving human participants were in accordance with the Declaration of Helsinki (as revised in 2013).

### Data analysis

Descriptive statistics (frequencies and percentages) were employed to describe the sociodemographic features of the participants. Afterward, chi-square (χ^2^) tests were used to test the relationships between potential factors and willingness to contract with family doctors in rural residents. Multivariable logistic regression was conducted to explore determinants of willingness to contract with family doctors. We incorporated the factors with *P* < 0.2 in the results of χ^2^ into the logistical model. All statistical analyses were conducted using STATA version 16.0 (Statacorp, College Station, TX). Statistical significance was set at *p* < 0.05.

## Results

### Characteristics of participants

A total of 769 unsigned rural patients with chronic diseases were included in the analysis. Table 1 shows the characteristics of the samples and the differences among rural residents’ willingness to contract with family doctors. Among the 769 participants, 46.7% (*N* = 359) were willing to contract with family doctors, and 53.3% (*N* = 410) were unwilling to contract with family doctors. Of the 769 participants, 45.5% were elderly (≥ 65 years), 58.0% were female, 35.8% were illiterate, 70.4% were employed, 83.2% were married, 98.3% had medical insurance, 39.6% reported fair self-rated health status, and 72.1% had at least one chronic disease. Approximately 77.5% of participants responded that they did not know about FDCS. Participants who stated that no one has ever publicized family doctor contract services accounted for approximately 84.5%. A total of 77.5% of the participants reported their need for utilizing chronic disease management programs, and 58.0% of them reported their need for utilizing referrals to specialists. Chi-square tests showed that there were statistically significant differences in gender, awareness of FDCS, publicity of FDCS, need for utilizing referrals to specialists, need for utilizing chronic disease management programs, and need for higher MI reimbursement rates.

**Table 1:** Characteristics of unsigned rural patients with chronic diseases in Shandong, China, 2018 (*N* = 769)

Characteristics	Frequency (%)	Willingness to contract with FDs^a^		*P*
		No	Yes	
**Observations**	769(100.00)	410(53.32)	359(46.68)	
**Gender**				0.008
Male	323(42.00)	154(47.68)	169(52.32)	
Female	446(58.00)	256(57.40)	190(42.60)	
**Age (years)**				0.131
< 65	419(54.49)	213(50.84)	206(49.16)	
> =65	350(45.51)	197(56.29)	153(43.71)	
**Education level**				0.121
Illiterate	275(35.76)	157(57.09)	118(43.00)	
Primary school	254(33.03)	140(55.12)	114(44.88)	
Junior middle school	182(23.67)	87(47.80)	95(52.20)	
Senior high school or above	58(7.54)	26(44.83)	32(54.69)	
**Employment status**				0.452
Unemployed	220(28.61)	122(55.45)	98(44.55)	
Employed	549(71.39)	288(52.46)	261(47.54)	
**Marital status**				0.667
Married	640(83.22)	339(52.97)	301(47.03)	
Single/divorce/widow	129(16.78)	71(55.04)	63(44.96)	
**Health insurance**				0.549
None	13(1.69)	8(64.29)	5(35.71)	
Have	756(98.31)	402(53.55)	354(46.45)	
**Household income**^b^				0.857
Q1	195(25.42)	100(51.28)	95(48.72)	
Q2	189(24.64)	103(54.50)	86(45.50)	
Q3	194(25.29)	101(52.06)	93(47.94)	
Q4	189(24.64)	104(55.03)	85(44.97)	
**Distance to the village clinic**				0.147
0–199 m	214(27.83)	128(59.81)	86(40.19)	
200–399 m	219(28.48)	108(49.32)	111(50.68)	
400–599 m	159(20.68)	82(51.57)	77(48.43)	
More than 600 m	177(23.02)	92(51.98)	85(48.02)	
**Awareness of FDCS**^c^				0.006
Never heard of it	596(77.50)	336(53.84)	260(46.16)	
Know little about it	147(19.12)	64 (43.54)	83(56.46)	
Know it well	26(3.38)	10(38.46)	16(61.54)	
**Publicity of FDCS**				<0.001
No	650(84.53)	365(56.15)	285(43.85)	
Yes	119(15.47)	45(37.82)	74(62.18)	
**Self-rated health status**				0.432
Good	209(27.18)	106(50.72)	103(49.28)	
Fair	305(39.66)	160(52.46)	145(47.54)	
Bad	255(33.16)	144(56.47)	111(43.53)	
**The number of chronic diseases**				0.593
1	530(72.14)	286(53.96)	244(46.04)	
> =2	239(27.86)	124(51.88)	115(48.12)	
**Referrals to specialists**				<0.001
No	458(59.56)	303(66.16)	155(33.84)	
Yes	311(40.44)	107(34.41)	204(65.59)	
**Chronic disease management programs**				<0.001
No	446(58.00)	314(70.40)	132(29.60)	
Yes	323(42.00)	96(29.72)	227(70.28)	
**Higher MI reimbursement rates**^d^				<0.001
No	491(57.23)	341(69.45)	150(30.55)	
Yes	367(42.77)	120(32.70)	247(63.70)	

*Note*: ^a^ FDs indicates family doctors; ^b^ Quartile 1 (Q1) is the poorest and Quartile 4 (Q4) is the richest; ^c^ FDCS indicates family doctor contract service; ^d^ MI indicates medical insurance

### Factors associated with participants’ willingness to contract with family doctors

Table 2 shows the determinants of willingness to contract with family doctors. For the enabling factors, residents who lived a greater distance from the village clinic (more than 600 m: OR = 1.85, 95% CI = 1.17–2.93) and received FDCS publicity (OR = 1.71, 95% CI = 1.06–2.76) had a significantly higher willingness to contract with family doctors. With the need factors, residents reporting the need for utilizing chronic disease management programs (OR = 3.36, 95% CI = 2.20–5.23) and the need for higher MI reimbursement (OR = 1.91, 95% CI = 1.28–2.83) were more likely to contract with family doctors.

**Table 2:** Multivariable logistic model of willingness to contract with family doctors among patients with chronic diseases in Shandong, China, 2018

Characteristics	Model	95%CI	***P***-value
	Odds Ratio		
**Gender**			
Male	1		
Female	0.73	0.50–1.05	0.088
**Age (years)**			
< 65	1		
> =65	0.96	0.68–1.35	0.805
**Education level**			
Illiterate	1		
Primary school	1.01	0.67–1.52	0.969
Junior middle school	1.29	0.80–2.09	0.299
Senior high school or above	1.04	0.51–2.11	0.909
**Distance to the village clinic**			
0–199 m	1		
200–399 m	1.41	0.92–2.17	0.117
400–599 m	1.57	0.98–2.52	0.058
More than 600 m	1.85	1.17–2.93	0.008
**Awareness of FDCS**^b^			
Never heard of it	1		
Know little about it	1.36	0.88–2.11	0.163
Know it well	1.10	0.43–2.87	0.838
**Publicity of FDCS**			
No	1		
Yes	1.71	1.06–2.76	0.028
**Referrals to specialists**			
No	1		
Yes	1.38	0.92–2.07	0.119
**Chronic disease management programs**			
No	1		
Yes	3.36	2.20–5.23	< 0.001
**Higher MI reimbursement rates**^c^			
No	1		
Yes	1.91	1.28–2.83	0.001

Note: ^a^ FDCS indicates family doctor contract service; ^b^ MI indicates medical insurance

*P < 0.05, **P < 0.01, ***P < 0.001

## Discussion

In recent years, the Chinese government has devoted more resources to promoting FDCS, and factors that may influence contract willingness are therefore significant to their implementation. Based on the Andersen model, this study explores the factors associated with of willingness to contract with family doctors for patients with chronic diseases living in rural areas. The role of need factors in affecting patients’ willingness to contract with family doctors was emphasized. The findings of this study will contribute to encouraging unsigned patients to contract with family doctors, to achieve the goal of FDCS system coverage.

In this study, only approximately 46.7% of unsigned rural patients with chronic diseases were willing to contract with family doctors. Residents are more willing to accept family doctors in developed countries, such as the United States (94%) [31] and Germany (74%) [32]. Thus, measures should be taken to promote their willingness to contract. In China, this rate was similar to another survey in Luzhou in 2019, where 46.62% of unsigned residents wanted to contract with family doctors [33]. It was lower than that in Xuzhou in 2018 (57.84%) [12] and in Henan Province in 2017 (74.5%) [25]. A possible explanation for this difference was that we only focused on residents in rural areas, and most of the previous studies were in urban areas. In general, rural residents had significantly lower willingness to sign contracts than those in urban areas [25]. In China, a shortage of well-trained and qualified village doctors, particularly in rural areas, is one of the main barriers to family doctor visits for residents [34]. Data from the China Health Statistics revealed that there were only, on average, 1.3 licenced physicians per thousand people in rural areas in 2018 [35]. The precondition for residents’ willingness to enroll is their trust in the quality of primary care and competency of family doctors. Hence, measures should be taken to increase the competence of primary health-care institutions, especially in rural China.

For enabling resources, it was interesting that residents who lived a further distance from the village clinic were more likely to contract with family doctors, which is contrary to previous studies [36]. In general, the spatial accessibility of healthcare is lower in rural areas, which increases rural residents’ time spent travelling and decreases their opportunities to visit family doctors. It seems that they are unwilling to contract with family doctors. However, family doctors can provide home visits for free, and residents whose homes are far away from the village clinic can obtain free door-to-door visits when needed. Accordingly, it is not difficult to understand why they were more likely to contract.

This study indicated that residents who received FDCS publicity were more likely to sign contracts, which is consistent with previous studies [24]. More publicity of FDCS provides residents more details about family doctors, and their potential benefits. Although the Chinese government has made efforts to promote the policy of family doctor service, residents in rural areas still have limited knowledge of the policy [37]. Rural residents generally have a low level of education and know little about the concept of a “family doctor”. Therefore, targeted publicity should be strengthened for chronically ill populations to improve their willingness to sign contracts.

The significant role of need factors on residents’ willingness to contract with family doctors was found in this study. We found that residents who reported their need for utilizing chronic disease management programs or the need for higher MI reimbursement rates were more willing to contract with family doctors. Residents with chronic diseases have a high need for healthcare services, if patients who contract with family doctors can receive more healthcare services, such as chronic disease management programs and medical services with higher reimbursement rates, they will be willing to sign contracts. Therefore, targeted services should be provided to improve their willingness to sign contracts.

There are several limitations in this study. First, the data we used were cross-sectional, making it impossible to draw causal inferences but only associations, and further studies are needed to explore the causal relationships. Second, the information used in this study was measured by self-reported information, which might result in recall bias. Finally, other factors, such as relationship with doctors, and satisfaction of participants, were not considered, which might also be significant determinants of willingness to enroll. Despite these limitations, we only focused on patients with chronic diseases living in rural areas, which is significant for making targeted recommendations for improving the implementation of FDCS in rural China.

## Conclusion

The prevalence of willingness to contract with family doctors was relatively low among unsigned rural patients with chronic conditions in rural Shandong, China. Factors including the distance to village clinics, publicity of FDCS, need for utilizing chronic disease management programs, and need for higher medical insurance reimbursement rates were associated with contract willingness. This study demonstrated that need factors were powerful factors explaining residents’ willingness to contract with family doctors. Therefore, to promote their willingness to contract, the government should strengthen targeted publicity to rural residents with chronic conditions. In addition, targeted services should be provided to improve their willingness to sign contracts.

## Supplementary Information

**Additional file 1: Appendix Figure 1** Andersen Model of factors influencing willingness to contract with family doctors in rural residents with chronic diseases.

**Additional file 2: Appendix Figure 2** Flow chart of sample selection.

## Data Availability

The datasets used and/or analysed during the current study are available from the corresponding author (Prof. Chengchao Zhou) upon reasonable request.

## Abbreviations

FDCS: Family doctor contract services
CDMP: Chronic disease management program
MI: Medical insurance
