# Supplementary material for: Willingness of patients with chronic disease in rural China to contract with family doctors: implication for targeting characteristics
Source: BMC Fam Pract. 2021 Oct 14;22:203. doi: 10.1186/s12875-021-01553-2 (PMC8518214; doi:10.1186/s12875-021-01553-2)
Supplement: Supplementary file 1 — Additional file 1: Appendix Figure 1 Andersen Model of factors influencing willingness to contract with family doctors in rural residents with chronic diseases. [file 12875_2021_1553_MOESM1_ESM.docx]

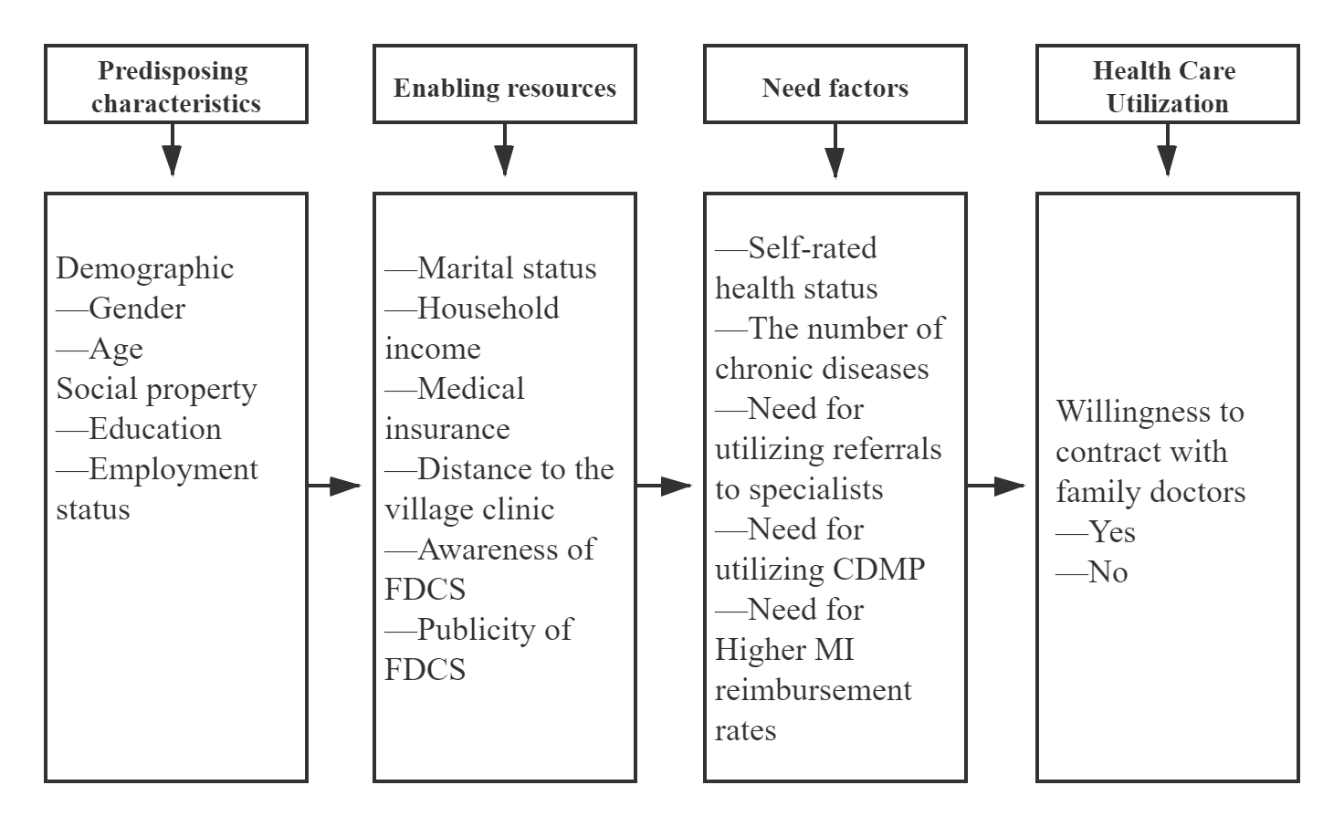


*Note: FDCS indicated family doctor contract services; CDMP indicated chronic disease management program; MI indicated medical insurance.*

**Appendix Figure 1** Andersen Model of factors influencing willingness to contract with family doctors in rural residents with chronic diseases
