# Supplementary figures and images for: Willingness of patients with chronic disease in rural China to contract with family doctors: implication for targeting characteristics
Source: BMC Fam Pract. 2021 Oct 14;22:203. doi: 10.1186/s12875-021-01553-2 (PMC8518214; doi:10.1186/s12875-021-01553-2)

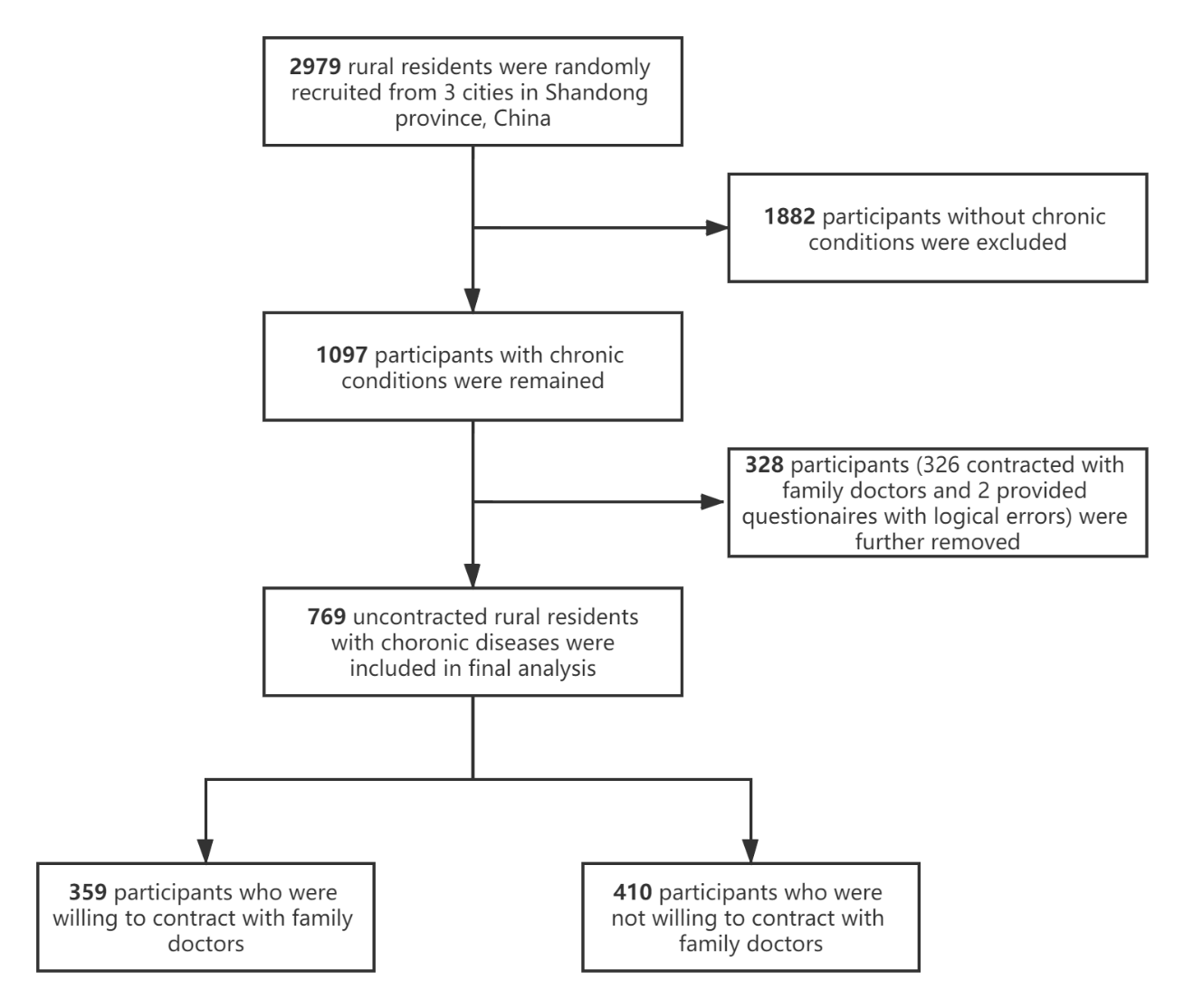


**Appendix Figure 2** Flow chart of sample selection

Supplement: Supplementary file 2 — Additional file 2: Appendix Figure 2 Flow chart of sample selection. [file 12875_2021_1553_MOESM2_ESM.docx]
